# Functional and clinical outcomes of FMRI-based neurofeedback training in patients with alcohol dependence: a pilot study

**DOI:** 10.1007/s00406-021-01336-x

**Published:** 2021-10-07

**Authors:** Susanne Karch, Daniela Krause, Kevin Lehnert, Julia Konrad, Dinah Haller, Boris-Stephan Rauchmann, Maximilian Maywald, Hessel Engelbregt, Kristina Adorjan, Gabriele Koller, Paul Reidler, Temmuz Karali, Nadja Tschentscher, Birgit Ertl-Wagner, Oliver Pogarell, Marco Paolini, Daniel Keeser

**Affiliations:** 1 Department of Psychiatry and Psychotherapy, University Hospital LMU, Nußbaumstr. 7, 80336 Munich, Germany; 2 Department of Radiology, University Hospital LMU, Munich, Germany; 3 Hersencentrum Mental Health Institute, Amsterdam, The Netherlands; 4 Institute of Psychiatric Phenomics and Genomics (IPPG), University Hospital, LMU, Munich, Germany; 5 Division of Neuroradiology, Department of Medical Imaging, The Hospital for Sick Children, University of Toronto, Toronto, Canada; 6 Munich Center for Neurosciences (MCN), LMU, Munich, Germany

**Keywords:** Real-time fMRI, Neurofeedback, Alcohol dependence, Addiction-associated brain responses

## Abstract

Identifying treatment options for patients with alcohol dependence is challenging. This study investigates the application of real-time functional MRI (rtfMRI) neurofeedback (NF) to foster resistance towards craving-related neural activation in alcohol dependence. We report a double-blind, placebo-controlled rtfMRI study with three NF sessions using alcohol-associated cues as an add-on therapy to the standard treatment. Fifty-two patients (45 male; 7 female) diagnosed with alcohol dependence were recruited in Munich, Germany. RtfMRI data were acquired in three sessions and clinical abstinence was evaluated 3 months after the last NF session. Before the NF training, BOLD responses and clinical data did not differ between groups, apart from anger and impulsiveness. During NF training, BOLD responses of the active group were decreased in medial frontal areas/caudate nucleus, and increased, e.g. in the cuneus/precuneus and occipital cortex. Within the active group, the down-regulation of neuronal responses was more pronounced in patients who remained abstinent for at least 3 months after the intervention compared to patients with a relapse. As BOLD responses were comparable between groups before the NF training, functional variations during NF cannot be attributed to preexisting distinctions. We could not demonstrate that rtfMRI as an add-on treatment in patients with alcohol dependence leads to clinically superior abstinence for the active NF group after 3 months. However, the study provides evidence for a targeted modulation of addiction-associated brain responses in alcohol dependence using rtfMRI.

## Introduction

Alcohol dependence is considered to be one of the most disabling mental disorders [1] and are difficult to treat [2, 3]. The incentive-sensitization model [4] of addiction describes that contexts of habitual drinking, or the smell of alcohol beverages, can turn into cues for alcohol consumption [5]. A meta-analysis demonstrated that alcohol cues elicited robust activation of the limbic and prefrontal cortex (PFC) in patients with alcohol dependence [6]. BOLD responses in these brain areas have been associated with emotional and reward-related processes [7]. Cue exposure therapy (CET) is a commonly used method in alcohol dependence [8–11]. However, CET alone has only shown inconsistent results in a meta-analysis [12].

Investigating the combination of cue exposure and its neurobiological correlates is more promising: The neurobiological mechanisms underlying substance abuse can be modulated with neurofeedback (NF) techniques based on real-time functional magnetic resonance imaging (rtfMRI). RtfMRI-NF provides a tool to monitor and self-regulate current brain activation [13–16] or brain connectivity patterns [17, 18]. Therefore, the rtfMRI-NF can be used to change brain activity associated with cognition or behaviour [19–23]. Almost all rtfMRI-NF studies of a recent review showed the ability to voluntarily modify signals in specific brain areas without determining their clinical relevance [24]. So far, there are only few studies focusing on NF processes in persons with addiction-related symptoms [25]. One of the first studies investigated the modulation of reward-related striatal brain responses in heavy social drinkers: A significant downregulation of striatal activation was observed in the active group [26]. For patients with alcohol dependence another study suggested that it is feasible to reduce their neuronal activity using rtfMRI NF [27]. Almost all studies of a recent rtfMRI review showed the ability to voluntarily modify signals in specific brain areas without determining its clinical relevance [24].

However, it is still unclear whether rtfMRI-NF leads to meaningful clinical improvements in patients with alcohol dependence. Double-blind placebo-controlled studies are needed to investigate its clinical relevance. Our hypothesis was that alcohol craving in patients can be altered on a clinically significant level using rtfMRI NF as an add-on to the standard treatment. In a double-blind, placebo-controlled setting we evaluated effects of clinical significance, 3 months after the last NF training. As a secondary aim, we investigated potential differences in neurobiological correlates between individuals without and with recurrence.

## Methods

### Study population

The study protocol was approved by the Ethics Committee at the Medical Faculty of Ludwig-Maximilians-University Munich, Germany. All patients provided written informed consent prior to their participation, in accordance with the Declaration of Helsinki and subsequent revisions. Participation in the study has not influenced treatment strategies; the participation in the NF study was an add-on to standard therapies. Each participant received 60€ per session.

Fifty-two patients (45 male; 7 female) were recruited from a specialised inpatient ward and specialised day hospital for patients with addiction disorders at the Ludwig-Maximilians-University Munich, Germany. Four male patients were excluded due to incidental brain findings or technical issues. Data of 48 patients (41 male; 7 female) were analyzed. The patients fulfilled the criteria for alcohol dependence according to DSM-IV and ICD-10. The age range was between 18 and 70 years. Exclusion criteria were substance abuse or withdrawal due to a substance other than alcohol (except tobacco); neurological or mental disorders other than alcohol dependence; any contraindication for MRI.

**Fig. 1:**
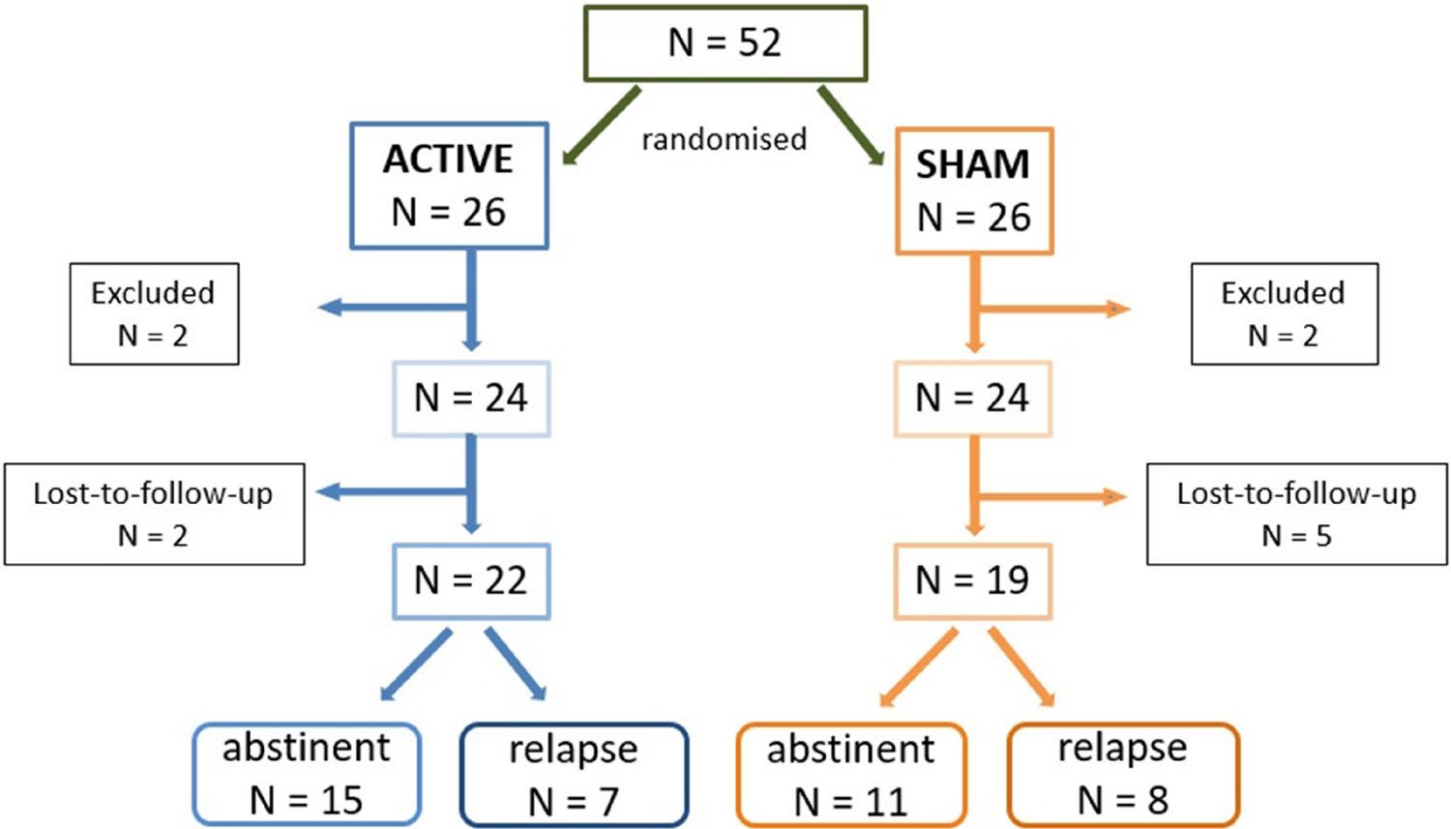
Experimental protocol. Active and sham rtfMRI conditions were applied in random order after baseline fMRI scans within a double-blind parallel group design

Participants were randomised in a double-blind manner into an active NF training group (*n* = 24; male = 19; female = 5; mean age = 45.1 years) and a sham NF group (*n* = 24; male = 22; female = 2; mean age = 45.9 years) (see Fig. 1 and Table 1). Patients of the active and the sham group did not differ regarding age (*p* = 0.816) and years of education (*p* = 0.247). Table 2 displays details about the duration of the treatment.

**Table 1:** Characteristics of participants

	Real group	Sham group
Total participants enrolled	26	26
Number of participants excluded	2	2
Female	5	2
Male	19	22
Age in years	45.1 (SD 13.3)	45.9 (SD 9.7)
Participants lost to follow-up	2	5
Abstinent patients	15	11
Relapse patients	7	8

*SD* standard deviation

**Table 2:** Duration of therapy

	Group	Mean	SD	*n*
Abstinent	Sham	41.60	22.94	10
	Real	23.53	11.78	15
	Total	30.76	18.97	25
Relapse	Sham	27.83	11.90	6
	Real	28.25	23.93	4
	Total	28.00	16.42	10
Total	Sham	36.43	20.26	16
	Real	24.52	14.40	19
	Total	29.97	18.09	35

*SD* standard deviation, *n* number

### Procedure of the neurofeedback fMRI

Participants underwent three NF sessions with 1–2 weeks between sessions. Psychometric assessments were performed before and after sessions. The total course of the study was 3–6 weeks, followed by the evaluation of abstinence 3 months later.

**Fig. 2:**
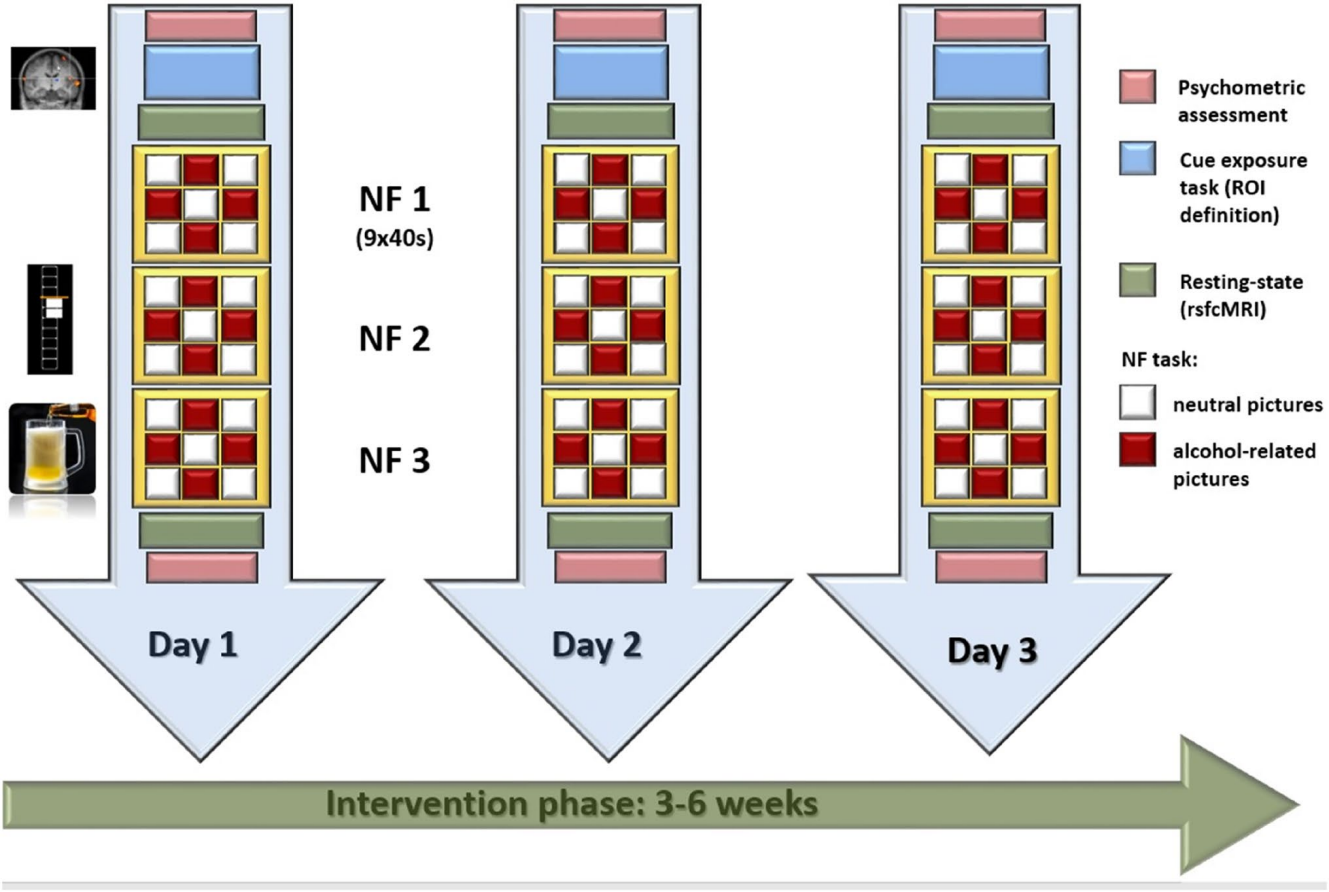
Experimental procedures: patients participated in three rtfMRI NF sessions within 3–6 weeks; during the NF training neutral and alcohol-related pictures were presented in blocks of 40 s with 10 pictures of the respective category; participants were instructed to reduce brain activity during the presentation of alcohol-associated information; during the presentation of neutral information, participants were instructed to simply gaze at the pictures. A cue exposure task was used to define the individual target regions (ROIs). Before and after each NF training resting-state activity was acquired and a psychometric assessment was performed. NF: neurofeedback

The following fMRI measurements have been performed: (i) cue exposure, (ii) resting state, (iii) rtfMRI NF paradigm (see Fig. 2).

(i) *Cue exposure* Participants saw pictures with either neutral or alcohol related cues using PsychoPy (v1.78.00, J. [28]). The neutral images were photographs of landscapes or objects from everyday life settings. The alcohol related pictures contained photographs of beer or wine and scenes with alcoholic drinks. Participants were instructed to look at the pictures. Craving related neuronal responses during the alcohol condition compared to the neutral condition were assessed. The cue exposure task was used to identify the target ROI for the NF training.

(ii) *Resting state* All participants were asked to close their eyes and stay awake without thinking of something in particular while a 7.5 min resting state-sequence (rsfcMRI) was acquired before and after each rtfMRI NF session.

(iii) *rtfMRI NF-paradigm* Throughout the NF sessions, neuronal responses were presented parallel to alcohol-associated pictures (cues) or neutral pictures. Each NF training session consisted of three runs. One NF run included 9 blocks of 40 s each. During 5 blocks neutral images were shown and during the other 4 blocks alcohol-related images were displayed (each for one second). Patients were told to reduce their individual neuronal responses in the target ROI while seeing the alcohol-related stimuli. ROI-based BOLD responses were measured and visualised using the Turbo-BrainVoyager (http://www.brainvoyager.com/TurboBrainVoyager.html). A graphical ‘thermometer’ based on the top one-third of voxels with the highest *t*-values for BOLD responses in the target ROI was utilised. During breaks, the perceived success of the participants during the rtfMRI training run was evaluated and everyone received feedback.

The total number of NF training sessions was three.

To ensure the double-blind design, there were always two examiners present during the NF session: One was responsible for the communication with the patients, the other one chose the region for modulation in accordance to active or sham.

### Definition of the target ROIs

The patients of the active group received feedback on their activation status for one of the following previously identified regions that highly correlated with craving in alcohol dependence: ACC [29–31], dorsolateral prefrontal cortex (DLPFC) [31, 32] and insula [27, 33]. Since neuronal response patterns across regions varied, the precise region used for NF was chosen according to the highest activation in the ACC, DLPFC or insula during cue exposure task. The size of the ROIs differed corresponding to the individualized brain activities. For that purpose, the percentage of activated voxel within each ROI (alcohol-associated pictures > neutral pictures) as well as the mean activation within each ROI has been calculated. Table 3 shows the chosen ROIs separately for patients of the active group that had remained abstinent 3 months after the NF training and those with a relapse.

**Table 3:** ROIs chosen for each NF session in REAL condition

	Abstinent [%]	Relapse [%]
Insula R	14.6	8.3
Insula L	14.6	16.7
DLPFC R	31.7	8.3
DLPFC L	19.5	16.7
ACC R	12.2	16.7
ACC L	7.3	33.3

For patients of the sham group an individual region located in the occipital was defined for the rtfMRI NF training. None of the chosen regions for the sham condition have been related to addictive behaviour or showed increased neural responses in the cue exposure task.

### Psychometric assessment

At the beginning of each NF session the following psychometric tests were used: Barratt Impulsiveness Scale (BIS-11) [34], Beck Depression Inventory (BDI) [35], State–Trait–Anger Expression Inventory (STAXI)[36], State–Trait–Anxiety Inventory (STAI) [37], obsessive compulsive drinking scale (OCDS) [38]. Additional assessments were conducted during the first session: socio-demographic data, the verbal intelligence test (WST) [39] and the NEO-Five Factor Inventory (NEO-FFI) [40].

### Evaluation of alcohol abstinence

During the treatment period, alcohol consumption was asked for and measured each time a patient returned to the ward, e.g. after examinations. The last assessment of the current alcohol intake was 3 months (± 4 days) following the last NF training and was done via telephone. Participants were asked to scale their motivation to remain abstinent or to document abstinence.

### Acquisition and analysis of fMRI

MRI imaging was performed using a Philips Ingenia 3 Tesla MRI scanner with echo planar capability (Release 4.1 Level 3 2013-04-05, Philips Medical Systems Nederland B.V.) and a 32-channel phased array head coil. The methods used for this study have been previously described [41]. To receive anatomical referencing, a T1-weighted high-resolution 3D data set was acquired for each participant. For functional BOLD imaging during the cue exposure and the rtfMRI-NF paradigms EPI sequences were obtained in the same position as the anatomical images (repetition time (TR): 2000 ms; echo time (TE): 35 ms; 25 axial slices; field of view: 230 mm × 230 mm; in-plane resolution: 3 mm × 3 mm; slice thickness: 4 mm; gap: 0.15 mm, flip angle (FA): 90°, number of scans: 180).

TurboBrainVoyager (Version 3.0, Brain Innovation, Maastricht, Netherlands) was used for primary processing and real-time analysis and also for the feedback to the participants. Additionally, raw-data in a DICOM-format were converted into a NIfTI-format using MRIConvert (Version 2.0.7 build 369, University of Oregon, Lewis Center for Neuroimaging, 2013). The BrainVoyager software package (Brain Innovation, Maastricht, Netherlands) was used for subsequent post-processing of data and analysis of the fMRI data. Because of relaxation time effects, the first five images of each run were not included in the analysis. Functional data were prepared together with high-pass filtering (cut-off: 3 cycles in a time course) to low-frequency signal drifts inherent in echo planar imaging, a spatial correction (cubic and trilinear interpolation), a slice scan time correction, spatial smoothing (Gaussian filter with FWHM 8.0 mm), and a 3D motion correction. Functional images were conveyed to a standard Talairach brain. A cross correlation of MR image pixel intensity with an expected hemodynamic response function was used to define a significant BOLD activity. Voxelwise *t* tests were performed to identify the areas where the signal change significantly differed between neutral and alcohol-related stimuli. The conditions neutral and alcohol-related images were considered as regressors for each patient.

All presented fMRI results were Bonferroni corrected. Only clusters exceeding the number of 30 voxels were considered.

### Statistical analysis

The statistical analysis was performed using SPSS (Version 24). All questionnaire data were analyzed using *t* tests for independent and paired samples or general linear models (significance: *p* < 0.05). A linear model (repeated measure method) was used for the comparison of variations before and after the NF training. Significant p values were Bonferroni corrected.

## Results

### Clinical data

Three months after the conclusion of the NF-training, 15 patients of the active group had remained abstinent, 7 had relapsed, 2 lost to follow-up (abstinence rate: 62.5%). In the sham group, 11 patients had remained abstinent, 8 had relapsed, 5 lost to follow-up (abstinence rate: 44%). No significant difference between the time frame from T1 to T2 (*p* = 0.288) and the time frame between T2 and T3 (*p* = 0.706) between patients that remained abstinent and patients with a relapse 3 months after the intervention was identified (Table 4). The abstinence rate did not differ significantly between groups (*p* = 0.247).

**Table 4:** Comparison of time frames between NF sessions (T1, T2, T3)

	T 1–T 2		T 2–T 3	
	*M*	SD	*M*	SD
Abstinent	7.96	2.60	8.13	2.74
Relapse	7.09	0.83	8.63	4.44

*M* mean, *SD* standard deviation

#### Verbal intelligence and personality

Participants did not differ regarding verbal intelligence (*p* = 0.371) and NEO-FFI factors (neuroticism: *p* = 0.665; extraversion: *p* = 0.644; openness to experience: *p* = 0.858; agreeableness: *p* = 0.668; conscientiousness: *p* = 0.668).

#### OCDS ratings

The comparison of OCDS ratings revealed a significant main effect for the NF session (*F *(1.5; 78) = 7.489; *p* = 0.003). The interaction between NF session*group (active; sham) was not significant (*F *(1.5; 58) = 0.278; *p* = 0.691).

#### Depression

The comparison of the BDI ratings revealed a significant main effect for the NF session (*F *(1.6; 61.3) = 10.994; *p* < 0.001). Post hoc analysis revealed a significant decrease of the BDI score between session 1 and session 2 (*p* = 0.007) as well as between session 1 and session 3 (*p* = 0.002). The interaction between NF session*group was not significant (*F *(1.6; 61.3) = 2.964; *p* = 0.071). Groups did not differ significantly (*F *(1; 39) = 0.004; *p* = 0.948).

#### Anxiety

Anxiety ratings did not differ between groups (STAI state: *F*(1; 40) = 0.019; *p* = 0.891; STAI trait: *F*(1; 37) = 0.170; *p* = 0.683) or sessions (STAI state: F (2; 80) = 0.514; *p* = 0.600; STAI trait: *F*(1.7; 62.1) = 3.296; *p* = 0.052). In addition, the interaction effects were not significant (STAI state; session*group: *F*(2; 80) = 0.158; *p* = 0.854; STAI trait; session*group: *F*(1.7; 62.1) = 0.765; *p* = 0.449).

#### Anger

Ratings of the anger control dimension differed significantly between groups with decreased scores in the active compared to the sham group (*F *(1;40) = 8.661; *p* = 0.005). Variations between sessions (*F *(2; 80) = 1.533; *p* = 0.222) as well as the interaction effect (session*group: *F*(2; 80) = 0.489; *p* = 0.615) were not significant.

#### Impulsiveness

Results of the non-planning subcategory of the BIS demonstrated significantly higher scores in the active group (*F* (1; 39) = 9.011; *p* = 0.005). Scores differed between sessions (*F* (2; 78) = 4.443; *p* = 0.015) with decreased scores on day 3 compared to day 1 (*p* = 0.030); the results of day 1 and day 2 (p = 1.000) as well as day 2 and day 3 (*p* = 0.082) did not differ. The interaction session*group were not significant (*F* (2; 78) = 0.347; *p* = 0.708).

In addition, all results concerning the subcategory motor impulsiveness (group: *F* (1; 39) = 2.255; *p* = 0.141; session: *F* (2; 78) = 1.314; *p* = 0.275; session*group: *F* (2; 78) = 0.944; *p* = 0.393) and attention to details (group: *F* (1; 40) = 2.026; *p* = 0.162; session: *F* (2; 80) = 1.536; *p* = 0.222; session*group: *F* (2; 80) = 0.005; *p* = 0.995) did not show significant differences.

### Neuronal responses

#### Comparison of neuronal responses before the NF training

*Active vs. sham* The comparison of BOLD responses between active and sham during the cue exposure task before the NF training did not show significant differences (alcohol-associated pictures compared to neutral pictures; fixed effects analysis, *q* (FDR) < 0.001, *p* (Bonf) < 0.001, *T* score: 5.6–8.0).

**Fig. 3:**
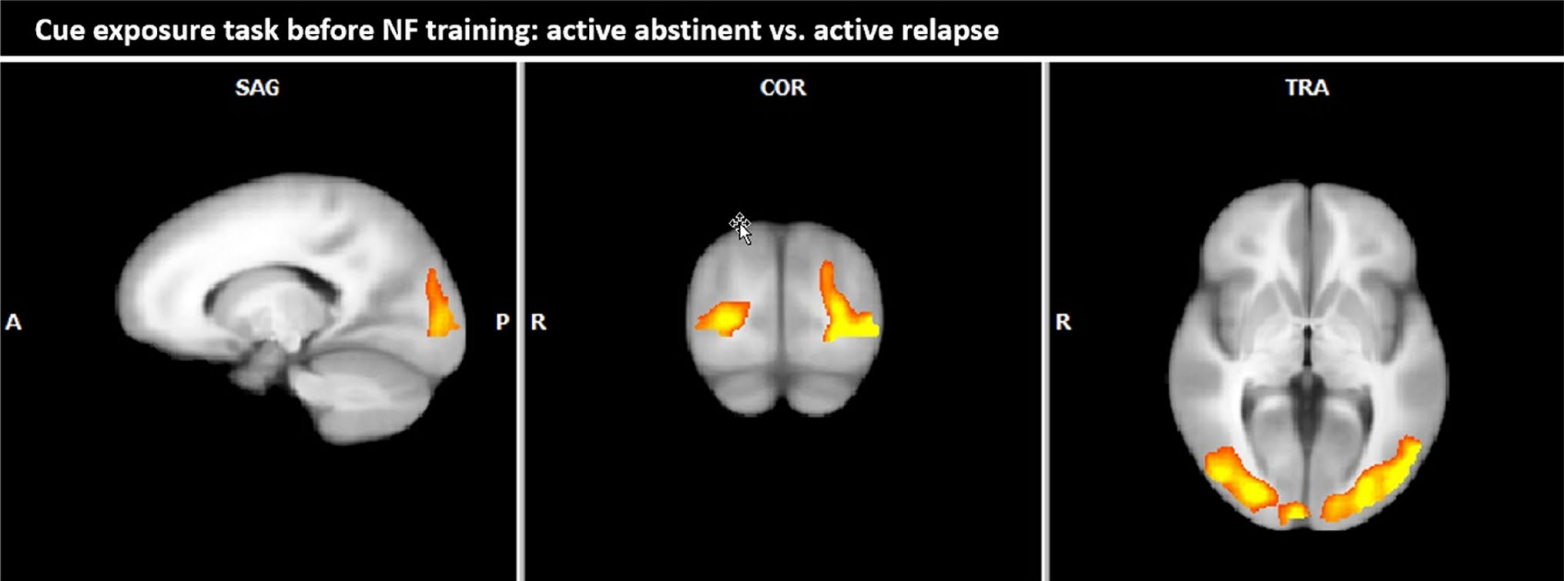
Comparison of craving-specific BOLD responses during active abstinent minus active relapse (alcohol-associated pictures minus neutral pictures) during the cue exposure task on day one (fixed effects analysis, p(Bonf) < 0.001, *T* score: 5.6–8.0, cluster threshold: 30 voxel; Talairach coordinates: *x* = -15, *y* = − 79, *z* = 0)

*Active abstinent vs. active relapse* During the cue exposure task prior to the NF training, patients of the active abstinent group demonstrated increased responses in the occipital cortex (see Table 5; Fig. 3).

**Table 5:** Comparison of BOLD responses during active abstinent minus active relapse (alcohol-associated pictures minus neutral pictures) during the cue exposure task on day one (fixed effects analysis, *p* (Bonf) < 0.001, *T* score: 5.6–8.0, cluster threshold: 30 voxel)

Brain region with increased responses during active abstinent compared to active relapse								
Brain region	BA	Side	Coordinates			Size	*T* score	
			TAL X	TAL Y	TAL Z		Ø	max
Occipital lobe								
Middle/inferior occipital gyrus	18/19	R	30	− 77	2	15,367	7.40	12.52
Middle/inferior occipital gyrus	18/19	L	− 29	− 78	1	8702	7.00	10.37

*BA* Brodmann area, *R* right, *L* left, *TAL X, Y, Z* Talairach-coordinates, *size* number of activated voxels, *max* maximum *T* score, *Ø* average *T* score, *NF* neurofeedback

#### Comparison of neuronal responses during the NF training

**Fig. 4:**
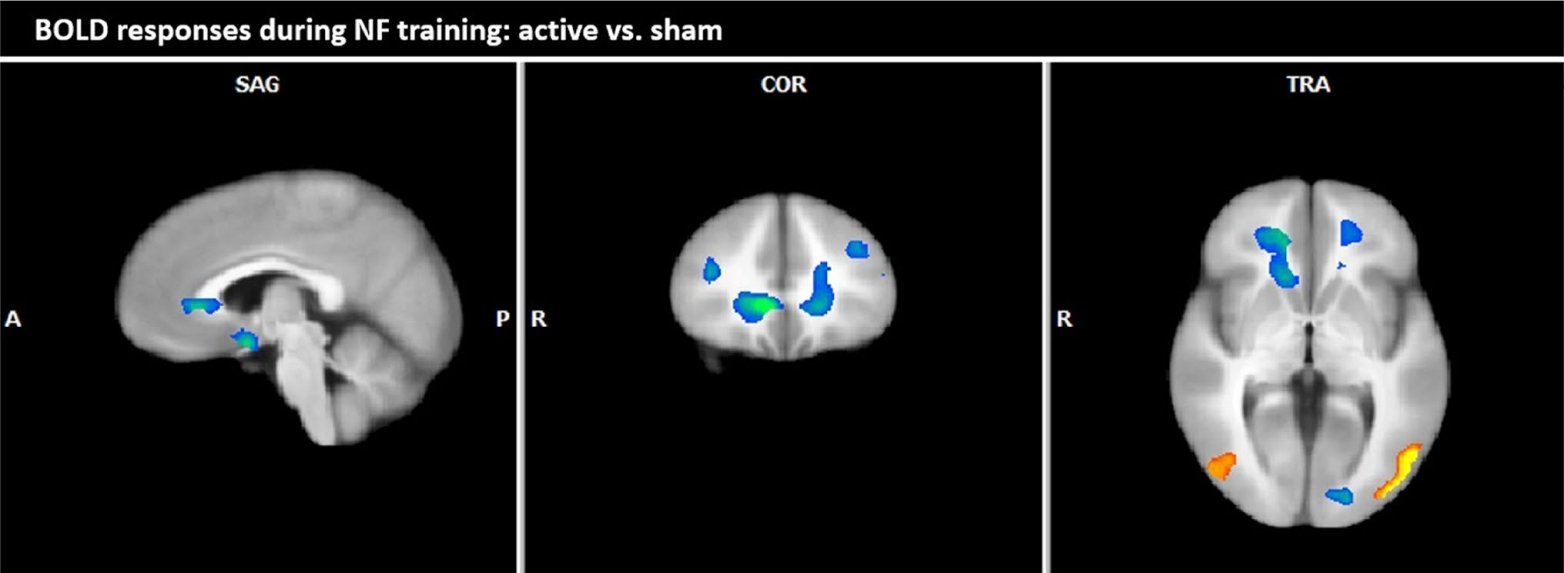
Overall group comparison active vs. sham: BOLD responses of the active group compared to the sham group across all NF sessions (contrast of alcohol-associated pictures vs. neutral pictures; fixed effects analysis, *p* (Bonf) < 0.001, *T* score: 5.6–8.0, cluster threshold: 30 voxel; Talairach coordinates: *x* = 4, *y* = 29, *z* = 0)

*Active vs. sham* The comparison of BOLD responses of the active and the sham group during NF training demonstrated decreased responses in the active group especially in medial frontal areas including the ACC and in the caudate nucleus. Brain responses in posterior areas, including the cuneus/precuneus and the inferior/medial occipital gyrus, were increased in the active group (see Table 6; Fig. 4).

**Table 6:** Overall group comparison active vs. sham: BOLD responses of the active group compared to the sham group across all three NF sessions (contrast of alcohol-associated pictures vs. neutral pictures; fixed effects analysis, *p* (Bonf) < 0.001, *T* score: 5.6–8.0, cluster threshold: 30 voxel)

Brain region	BA	side	Peak coordinates			size	*T* score	
			TAL X	TAL Y	TAL Z		Ø	max
Brain region with decreased BOLD responses during active compared to sham								
Frontal lobe								
Anterior cingulate cortex	24, 32	R	19	26	10	15,402	– 6.60	– 9.26
Anterior cingulate cortex/claustrum	24, 32	L	– 21	23	16	10,339	– 6.28	– 8.50
Caudate nucleus		R	19	26	10	15,402	– 6.60	– 9.26
Occipital lobe								
Lingual gyrus, cuneus	17	L	– 15	– 88	4	1513	– 6.96	– 9.22
Brain region with increased BOLD responses during active compared to sham								
Occipital lobe								
Cuneus, precuneus	18/31	R	20	– 76	19	1996	6.85	9.02
Inferior occipital gyrus	19	R	42	– 72	– 3	908	6.41	7.64
Medial occipital gyrus	19/37	L	– 45	– 72	4	3442	7.24	11.01
Temporal lobe								
Inferior temporal gyrus	20	R	44	– 12	– 16	2082	6.69	9.68
Medial temporal gyrus	39	R	52	– 57	10	1258	6.67	9.19

*BA* Brodmann area, *R* right, *L* left, *TAL X, Y, Z* Talairach-coordinates, *size* number of activated voxels, *max* maximum *T* score, *Ø* average *T* score, *NF* neurofeedback

**Fig. 5:**
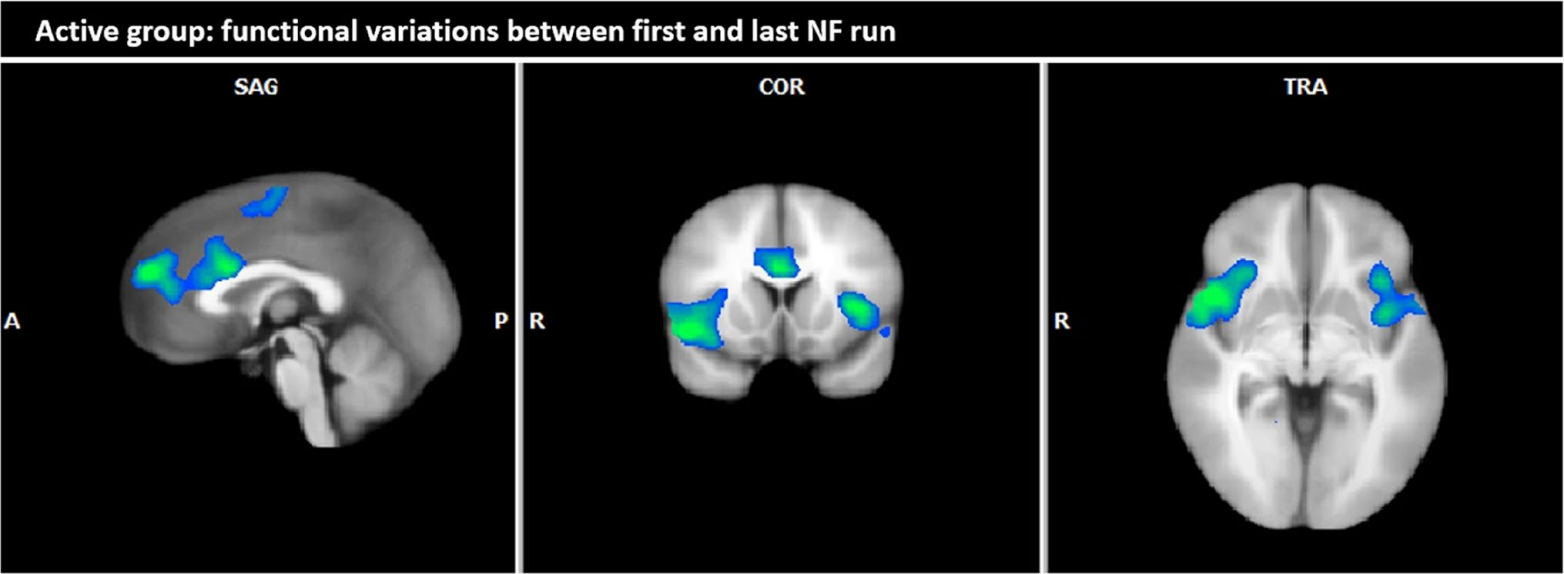
Overall within-active group comparison: BOLD responses 3rd NF run, 3rd session vs. 1st NF run, 1st session (contrast alcohol-associated pictures vs. neutral pictures; fixed effects analysis, *p* (Bonf) < 0.001, *T* score: 5.6–8.0, cluster threshold: 30 voxel; Talairach coordinates: *x* = 0, *y* = 13, *z* = − 3)

*Active* In patients of the active group, BOLD responses at the end of the NF training (3rd session, 3rd NF run) were decreased compared to the beginning of the NF training (1st session, 1st NF run) in the anterior part of the medial frontal gyrus, the ACC, the pre-/postcentral gyrus and the left and right insular cortex, especially in its anterior parts (see Table 7; Fig. 5).

**Table 7:** Overall within-active group comparison: BOLD responses 3rd NF run, 3rd session vs. 1st NF run, 1st session (contrast alcohol-associated pictures vs. neutral pictures; fixed effects analysis, *p* (Bonf) < 0.001, *T* score: 5.6–8.0, cluster threshold: 30 voxel)

Brain region with decreased BOLD responses during last NF run compared to the first NF run								
Brain region	BA	Side	Coordinates			Size	T score	
			TAL X	TAL Y	TAL Z		Ø	max
Frontal lobe								
Medial frontal gyrus	6	L/R	0	– 6	57	1600	– 6.01	– 6.74
Postcentral gyrus	3	R	47	– 12	42	1910	– 6.16	– 7.30
Precentral gyrus	44	L	– 40	10	2	9430	– 6.76	– 8.53
	4	R	47	– 12	42	1910	– 6.16	– 7.30
Insular cortex	13	L	– 40	10	2	9430	– 6.76	– 8.53
Subgyral		R	18	26	14	35,203	– 6.57	– 9.45
Cerebellum								
Culmen, declive		R	18	– 55	– 10	836	– 6.20	– 7.12
Temporal lobe								
Parahippocampal gyrus	19	R	18	– 55	– 10	836	– 6.20	– 7.12

*BA* Brodmann area, *R* right, *L* left, *TAL X, Y, Z* Talairach-coordinates, *size* number of activated voxels, *max* maximum *T* score, *Ø* average *T* score, *NF* neurofeedback

*Sham* In patients of the sham group, BOLD responses did not differ significantly between the end of the NF training (3rd session, 3rd NF run) and the beginning of the NF training (1st session, 1st NF run).

**Fig. 6:**
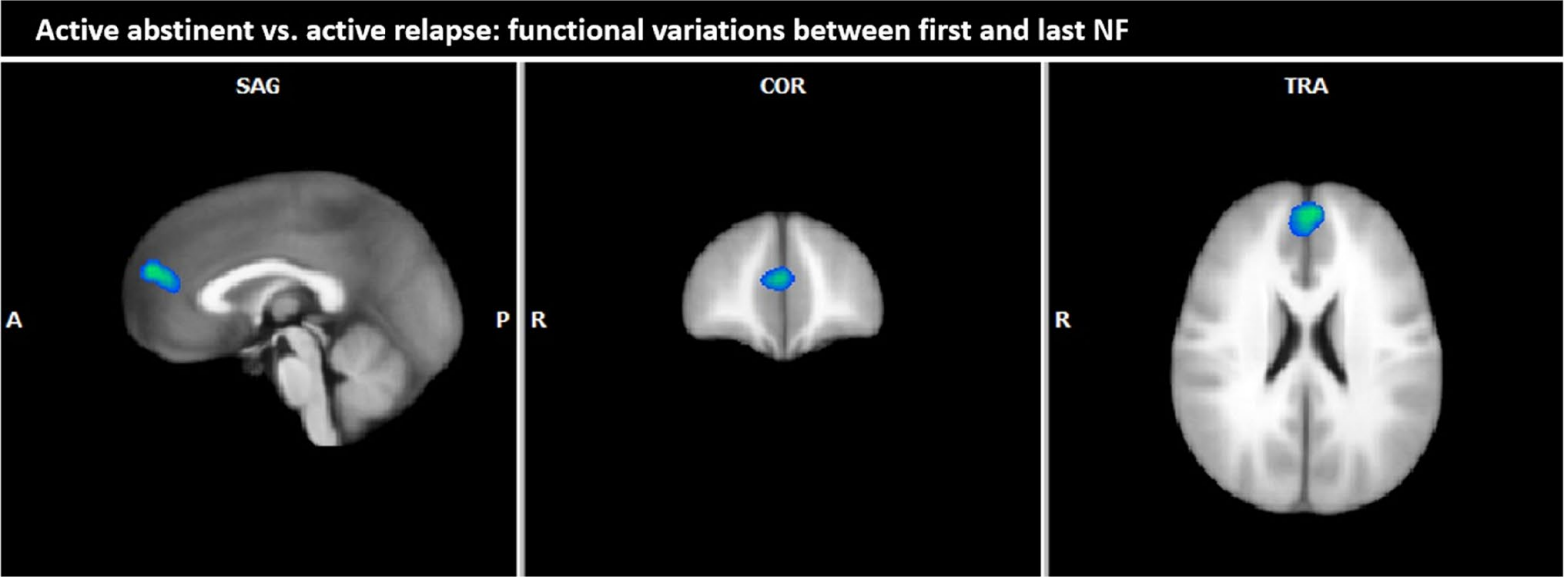
Within-active group comparison, abstinence vs. relapse (contrast alcohol-associated pictures compared to neutral pictures during the 3rd NF run minus 1st NF run of NF session 1, 2, 3; fixed effects analysis, *p* (Bonf) < 0.001, *T* score: 5.6–8.0, cluster threshold: 30 voxel; Talairach coordinates: *x* = 0, *y* = 45, *z* = 23)

*Active abstinent vs. active relapse* Patients of the active group who had remained abstinent for an interval of at least 3 months after the NF training have been able to reduce their neuronal responses during the NF training in the anterior part of the medial frontal gyrus more strongly than patients with a relapse within an interval of 3 months after the NF training (see Table 8, Fig. 6).

**Table 8:** Within-active group comparison, abstinence vs. relapse (contrast alcohol-associated pictures compared to neutral pictures during the 3rd NF run minus 1st NF run of NF session 1,2,3; fixed effects analysis, *p* (Bonf) < 0.001, *T* score: 5.6—8.0, cluster threshold: 30 voxel)

Brain region with decreased BOLD responses during active abstinent versus active relapse								
Brain region	BA	Side	Coordinates			Size	T score	
			TAL X	TAL Y	TAL Z		Ø	max
Frontal lobe								
Medial frontal gyrus	8/9	L/R	0	47	20	2510	− 6.42	− 7.97

*BA* Brodmann area, *R* right, *L* left, *TAL X, Y, Z* Talairach-coordinates, *size* number of activated voxels, *max* maximum *T* score, *Ø* average *T* score, *NF* neurofeedback

#### ROI fMRI analysis

For the ROI analysis, the number of activated voxels (alcohol-associated pictures > neutral pictures) percentual in comparison to the ROI size has been calculated. In addition, we calculated the mean activation within each ROI.

*Comparison of active vs sham group* The number of activated voxels as well as the mean activation within each ROI (alcohol-associated pictures > neutral pictures) did not show significant differences regarding session, group (active vs. sham) or interaction effects (session * group; NF run * group; session * NF run; session * NF run * group). In addition, group differences did not differ significantly.

*Comparison of active abstinent vs active relapse* The number of activated voxels (alcohol-associated pictures > neutral pictures) and the mean activation within each ROI did not show significant differences regarding session, group or interaction effects (session * group; NF run * group; session * NF run; session * NF run * group).

## Discussion

The goal of our double-blinded, sham-controlled study was to identify whether patients with alcohol dependence profit from craving-related NF training as an add-on to the standard treatment. We predicted a clinically desirable abstinence rate of at least 3 months after the training and a stronger neurobiological impact of the training in the group that remained abstinent.

### Comparison of clinical data of the active group with the sham group

In the clinical data, only slight alterations between the two groups were identified that did not reach a significant level. During the 3 months of the study period, the difference of the overall abstinence rates (active group: 62.5% vs. sham: 44%) was too small to proof a therapy effect of the rtfMRI-NF training. One reason for this may be that patients of both groups took part in the alcohol dependence standard treatment; the add-on effect of rtfMRI-NF could have been comparatively small taking into account the whole therapy program. For that purpose, the sample size of the study was probably undersized to outline differences between groups.

The assessment of the cognitive craving for alcohol remained stable over the course showing no difference between active and sham. The general association of craving and the final treatment success of abstinence remains a matter of debate: After cue exposure treatment, patients with alcohol dependence with higher craving had a lower 3-month rate of abstinence [42]. Others have suggested the opposite, and instead have identified a negative affective state as a main driver for relapse [43]. Our study identified a decrease of depressive symptoms from the first to the last NF session, although without a difference between active and sham.

### Comparison of neurobiological data of the active group with the sham group

The overall group comparison of patients with alcohol dependence revealed significantly reduced activity in the ACC, the claustrum, and the caudate nucleus in the active group in comparison to the sham group during the NF training over the whole study course. It is likely that the effects measured are mainly caused by the changes in the active group, as neurobiological data remained stable in the sham group. The claustrum and the caudate nucleus play a role in cognitive restoration processes especially conducted by interactions over the frontal association areas [44, 45]. The basal ganglia are part of neuronal circuits that play a role in alcohol dependence [46–48]. This indicates that a reduction of neuronal activity within these brain areas during the course of the NF training may influence addictive behaviour. For patients of the active group, a higher activity in the cuneus and parts of the occipital region were shown. These regions of the visual system are not specifically associated with addictive disorders. Yet, neuronal circuits from these visual information processing regions lead secondary to parietal, temporal and frontal regions associated with attention and memory functions, suggesting that this information from cues could be connected with already established content [44]. Similar findings of visual-emotional processing after presenting alcohol-associated cues have been demonstrated [49].

### Changes of neuronal activity in patients with alcohol dependence of the active group

Our main finding for the active group over the entire study period was the significant reduction of cue-associated neuronal activation in frontal brain regions. The reduction of neuronal activity was most prominent in the medial frontal gyrus, the dACC, the insula, and the precentral gyrus as well as in the neighbouring parietal areas. Previously these regions have already been identified as important in addictive disorders [50–52]. The dACC and the frontoinsular cotex bilaterally which are mainly concerned by BOLD signal reductions represent core parts of arousal processing and salience [53]. A decrease of neural activity in these areas may indicate less cue-induced bottom up salience during the NF by enhanced top down control with potential impact on behaviour. Our results showed a reduction in activity of the parahippocampal gyrus in the active group. This gyrus is part of the limbic system and involved in memory, learning and processing of emotions [44]. The limbic system is more activated in individuals with alcohol dependence after the consumption of alcohol [31]. Therefore, one interpretation of our activity reduction in the parahippocampal gyrus could be that well-rehearsed behaviour patterns of addiction had lost their relevance leading to a weaker association of new alcohol-related stimulus with stored memory. This effective possibility of downregulating neuronal activity in response to addiction-related stimuli with rtfMRI NF was demonstrated in the ACC, in frontal regions and the insula during cue exposure in patients with alcohol dependence [27].

Furthermore, we compared the neuronal activity of all active patients who had been abstinent for at least 3 months with the activity of relapsed patients. The significantly reduced neuronal activity in the anterior part of the medial frontal gyrus in the abstinent active group, especially in the medial prefrontal cortex (MPFC), was the main finding. Previous studies have identified the MPFC as a highly relevant region for cue associated neural responses and craving and therefore as a promising specific target for neuromodulation in alcohol dependence [54].

### Limitations

First, the individual level of motivation to participate in this study may have been influenced by the financial incentive (180€). Second, the nature of this study was exploratory and the number of participants in some subgroups such as abstinent versus relapse patients within the active group was partly underpowered. However, the total number of finally included patients was 48, which is relatively high considering the existing literature. Third, the male to female ratio was not balanced. Gender effects were calculated without showing a significant influence. As sex-specific differences can occur, it is recommended to validate the present results with larger sample sizes and an equal gender distribution. Fourth, respiratory artifacts were not taken into consideration during the data analysis [55] as well as the time frame between NF sessions. Fifths, the exact number of NF sessions to produce the most effective results still remains a matter of debate [41]. Furthermore, out of the four main brain regions associated with craving, only three were included in the individualized ROIs. The ventral striatum was only part of the whole brain analysis and would have been another brain region that could have been useful for modulation. However, up to now no studies exist that compared the efficiency of both strategies. Studies that compare the effectiveness of the modulation of brain activity in various brain regions would be helpful to get further insight into the underlying brain mechanisms for the ROI selection in patients with AUD.

## Conclusion

This double-blinded, sham-controlled study could not demonstrate that rtfMRI as add-on to a standard treatment in patients with alcohol dependence leads to a clinically superior abstinence rate for the active NF group compared to a sham group after 3 months . However, we found evidence for a targeted modulation of specific addiction-associated brain regions during rtfMRI NF. Abstinent patients of the active group demonstrated the most prominent ability to reduce their craving-related neuronal activity. Given the neurobiological effects, our results suggest that further studies with larger sample sizes are needed to prove the clinical benefit of rtfMRI NF for treating alcohol dependence. Evidence from further studies is in particular required concerning long-term effects in different subgroups.

## Data Availability

The data that support the findings of this study are openly available in OSF at https://osf.io/zv83b/
